# Potential of a Miniature Spectral Analyzer for District-Scale Monitoring of Multiple Gaseous Air Pollutants

**DOI:** 10.3390/s23146343

**Published:** 2023-07-12

**Authors:** Alaa Fathy, Martine Gnambodoe-Capochichi, Yasser M. Sabry, Momen Anwar, Amr O. Ghoname, Ahmed Saeed, Yamin Leprince-Wang, Diaa Khalil, Tarik Bourouina

**Affiliations:** 1 ESYCOM, UMR 9007 CNRS, Univ Gustave Eiffel, 77454 Marne-la-Vallée, France; alaa.fathy@esiee.fr (A.F.); martine.capo-chichi@univ-eiffel.fr (M.G.-C.); yamin.leprince@univ-eiffel.fr (Y.L.-W.); 2 Si-Ware Systems, Cairo 11361, Egypt or yasser.sabry@eng.asu.edu.eg (Y.M.S.); momen.anwar@si-ware.com (M.A.); amr.ghoname@eng.asu.edu.eg (A.O.G.); diaa_khalil@eng.asu.edu.eg (D.K.); 3 Faculty of Engineering, Ain-Shams University, Cairo 11535, Egypt; 4 CINTRA, IRL 3288 CNRS-NTU-THALES, Nanyang Technological University, Singapore 637553, Singapore

**Keywords:** air pollution, multi-parameter gas sensing, mid-infrared spectroscopy, open FTIR, MEMS

## Abstract

Gas sensors that can measure multiple pollutants simultaneously are highly desirable for on-site air pollution monitoring at various scales, both indoor and outdoor. Herein, we introduce a low-cost multi-parameter gas analyzer capable of monitoring multiple gaseous pollutants simultaneously, thus allowing for true analytical measurement. It is a spectral sensor consisting of a Fourier-transform infrared (FTIR) gas analyzer based on a mid-infrared (MIR) spectrometer. The sensor is as small as 7 × 5 × 2.5 cm^3^. It was deployed in an open-path configuration within a district-scale climatic chamber (Sense City, Marne-la-Vallée, France) with a volume of 20 × 20 × 8 m^3^. The setup included a transmitter and a receiver separated by 38 m to enable representative measurements of the entire district domain. We used a car inside the climatic chamber, turning the engine on and off to create time sequences of a pollution source. The results showed that carbon dioxide (CO_2_) and water vapor (H_2_O) were accurately monitored using the spectral sensor, with agreement with the reference analyzers used to record the pollution levels near the car exhaust. Furthermore, the lower detection limits of CO, NO_2_ and NO were assessed, demonstrating the capability of the sensor to detect these pollutants. Additionally, a preliminary evaluation of the potential of the spectral sensor to screen multiple volatile organic compounds (VOCs) was conducted at the laboratory scale. Overall, the results demonstrated the potential of the proposed multi-parameter spectral gas sensor in on-site gaseous pollution monitoring.

## 1. Introduction

Air pollution has become a growing concern worldwide, causing the deaths of seven million individuals annually, as reported by the World Health Organization [1]. This alarming situation highlights the need to develop new strategies and tools to monitor air pollution and improve air quality. Outdoor pollution, which is mainly attributed to human activities, particularly transportation, has a significant impact on indoor air quality. In addition to outdoor pollutants, volatile organic compounds (VOCs) also contribute to indoor air pollution. On-site outdoor gas monitoring is crucial for mapping pollution at different scales [2,3,4,5]. However, there is a rich diversity of air pollutants, especially regarding the numerous gaseous pollutant species of interest, which require the use of as many different kinds of sensors, resulting in sophisticated, bulky and expensive measurement systems. Although some multi-parameter sensors are commercially available [6,7], they are constructed via the assembly of different sensors and are limited to a very few types of pollutants; more importantly, the rather high cost restricts the number of location spots in a field deployment of pollution sensors. This technological limitation also restricts the diversity of pollutants that can be monitored [4,5]. Open-path Fourier-transform infrared (FTIR) spectroscopy is a relevant technique for such monitoring, as it allows for the measurement of multiple gaseous air pollutants using a single device. It is widely used to measure atmospheric traces and greenhouse gases, such as carbon dioxide and methane [8,9], study chemical emissions [10,11,12], and measure ozone levels [13] and volcanic emissions [14]. However, the benchtop spectrometers required for open-path FTIR measurements are expensive and bulky, limiting the installation process and wide deployment of a large number of such systems for pollution mapping or monitoring a given area. Therefore, a low-cost and miniaturized sensor capable of monitoring pollution would be a key enabling technology toward ubiquitous pollution monitoring. The low-cost potential of the proposed sensor is indeed expected from its core MEMS fabrication technology, which is already proven to enable large-volume manufacturing of sensors at low cost in many application areas including automobiles, smartphones, and consumer electronic products.

In this paper, we propose a low-cost and compact spectral sensor that can monitor multiple gaseous air pollutants using a single device. The proposed device is an open-path Fourier-transform infrared (FTIR) gas analyzer based on a mid-infrared (MIR) MEMS spectrometer, produced using MEMS technologies. Before the massive deployment of such spectral sensors, it is important to assess their potential in practical use cases. Therefore, the device was deployed inside a district-scale climatic chamber (Sense City, Marne-la-Vallée, France [15]) to demonstrate its capabilities in monitoring the air pollution produced by a car engine in this experiment. Furthermore, we present preliminary results of laboratory-scale measurements intended to assess the potential of the proposed spectral sensor to screen volatile organic compounds (VOCs) and determine their potential impact on health. For example, the recommended exposure limit (REL) to toluene is a concentration ranging from 20 ppm to a maximum of 100 ppm during 8 h according to the National Institute for Occupational Safety and Health (NIOSH). Therefore, the ability to distinguish toluene (and others) among all VOCs is essential.

## 2. Materials and Methods

### 2.1. Sense City: A District-Scale Climatic Chamber

**Figure 1:**
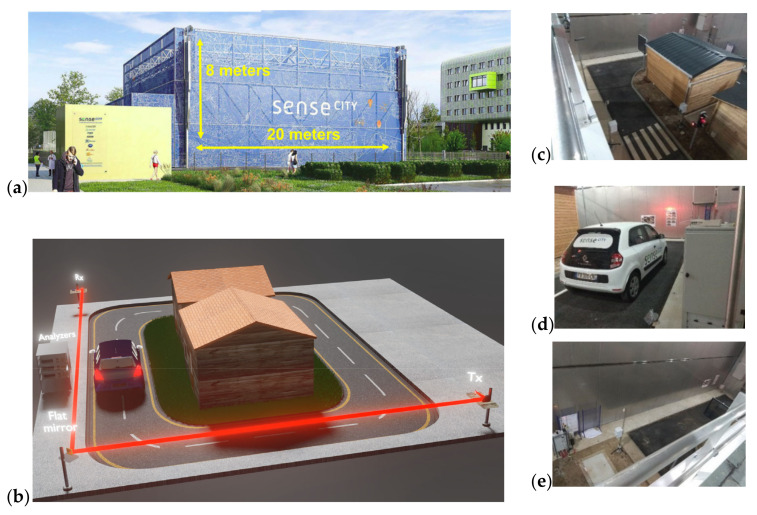
(**a**) The Sense City district-scale climatic chamber, (**b**) our experiment scenario, (**c**–**e**) illustrative photos.

Sense City is a climate chamber at the district scale with dimensions of 20 m × 20 m × 8 m. It is used to conduct various experiments under controlled environments; it has the capability to vary different parameters such as temperature, humidity, sunlight and rain. A variety of reference sensors are installed in Sense City, whose design aims to study different urban scenarios dealing with the broader topic of smart cities. These relate, for instance, to energy and environmental problems including air, water and soil. The Sense City climatic chamber is introduced in Figure 1 along with the scenario of our experimental setup deployed for monitoring multiple gaseous air pollutants within this area.

### 2.2. Experimental Setup

**Figure 2:**
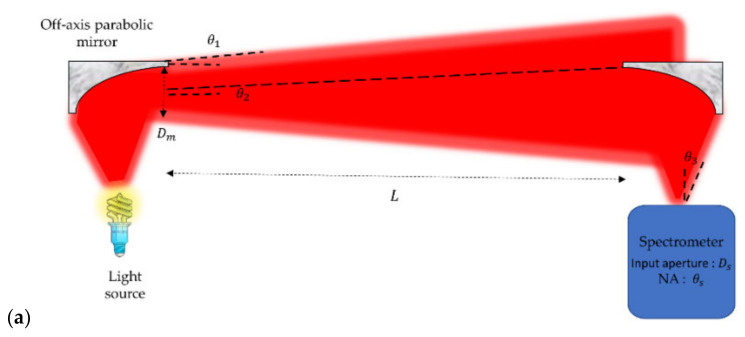

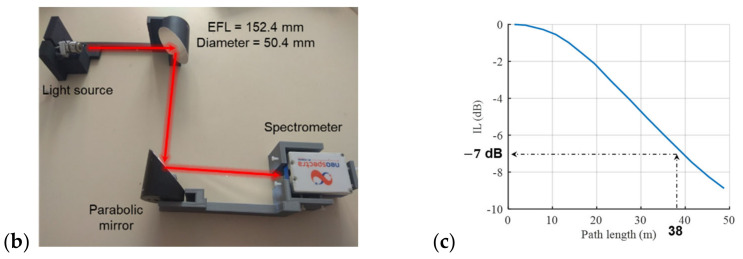
(**a**) Schematic layout of open-path setup using off-axis parabolic mirrors to transfer light from light source Tx to the spectrometer Rx, (**b**) camera photo of the optical system used in the open-path FTIR, (**c**) insertion loss (*IL*) of the coupling system as a function of the path length.

In the main experiment, the spectral gas sensor is built from a core element, which is a MEMS-based FTIR spectrometer (NeoSpectra MIR SWS6241, Si-Ware Systems, Cairo, Egypt). The version that we used in our experiments is a prototype with an extended wavelength compared to the Neospectra SWS62221 commercial product, which is limited to the near-infrared (NIR) from 1.3 to 2.5 μm. This prototype has a spectral range from 1.5 to 4.9 μm in the mid-infrared (MIR). Such an extended range covers the absorption spectral signatures of CO_2_, CO and NO_x_ as well as H_2_O and multiple volatile organic compounds (VOCs). The optical system used in the open-path FTIR measurements comprises a light source (also referred to as the transmitter, Tx, in Figure 1b). It consists of an infrared light bulb (temperature = 2796 K, SLS252, Thorlabs, Newton, NJ, USA) placed at the focal point of the off-axis parabolic mirror (diameter = 50.8 mm, effective focal length = 152.4 mm, MPD269-G01, Thorlabs), as shown in Figure 2a,b.

In a second experiment, we used a multi-pass gas cell (Tornado T20, Specac, Kent, UK) with a base length of 0.5 m, allowing for 40 reflections, and presenting a total effective range of 20 m. This allows the implementation of an alternative to open-path absorption in a more compact space.

The parabolic mirrors play a crucial role in minimizing the intrinsic insertion loss (*IL*) especially considering the rather long propagation distance of 38 m considered in our experiment. Indeed, the fundamental role of the parabolic mirror is to achieve light collimation, which will ensure that the light spot does not increase too much after light propagates over a long distance, as illustrated in Figure 2a, leading to limited insertion loss as shown in Figure 2c. The collimated light from the source is refocused at the detector side using another off-axis parabolic mirror, whose function is to transform the incident collimated light beam into a much smaller spot concentrated over the input window of the spectrometer. The light is focused into the input of the MIR spectrometer (also referred to as the receptor, Rx, in Figure 1b). The mechanical part holding different optical parts was designed using a CAD tool and made of plastic using 3D printing.

**Figure 3:**
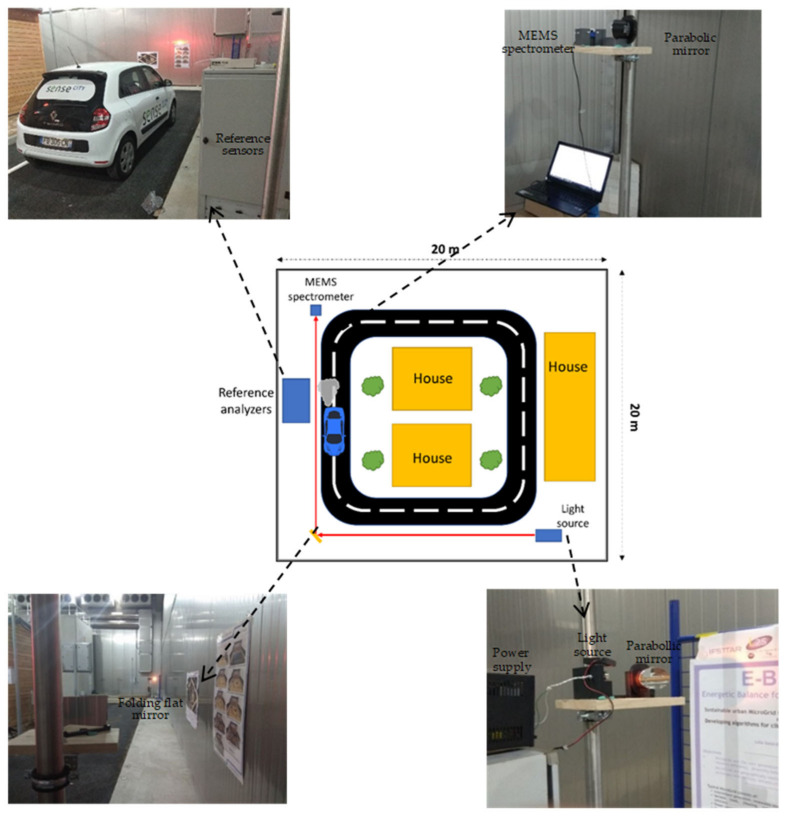
Top schematic view of Sense City for air pollution monitoring using open-path FTIR in addition to camera photos at different positions inside the city.

As shown in Figure 3, the light source is positioned on a stand at the bottom right corner of the chamber, while the MEMS spectrometer is placed at the top left corner. The collimated light propagates from the source along the road to the bottom left corner of Sense City. Then, it undergoes a 90° deflection by a flat mirror and passes near the car before reaching the top left corner. The total optical path from the source to the detector is *L* = 38.1 m. Such a rather large distance introduces optical insertion losses (*ILs*) of −7dB (Figure 2c). This signal loss is evaluated with the support of additional design considerations that are given in the following section. It is worth mentioning that the optical losses considered at this stage are *intrinsic losses* that are independent of the local environmental gaseous contents. Besides those intrinsic losses, there will be additional losses that are optical *absorption losses* due to the gaseous pollutants. These absorption losses are dependent on the local environment. The spectrum and the magnitude of these absorption losses depend on the types of gases and their corresponding concentrations, respectively.

While the reference analyzer measures the local gas concentration near the car, the open-path FTIR measures an average gas concentration along the distance *L*. High-performance analyzers are installed in Sense City. They provide reference measurements on the concentrations of nitrogen oxide NO_x_, CO and CO_2_. Additional reference sensors are used for humidity and temperature measurements. The NO_x_ analyzer (AC32M, ENVEA) has a lower limit of detection of 0.5 ppb. The CO analyzer (CO12e, ENVEA) has a lower limit of detection of 25 ppb. The analyzer has an additional module for CO_2_ measurement. Both reference gas analyzers were installed beside the car. They measure the gas concentration while sucking in local air.

## 3. Results and Discussion

### 3.1. Design Considerations on the Setup for Open-Path Gas Absorption Spectroscopy

In radiometry, the light power emitted from a circular source can be given by [16]:

(1) $$P=\int_{0}^{\pi {\left(0.5D\right)}^{2}}\int_{0}^{2\pi }\int_{0}^{\theta_{o}}R_{o}\;cos\theta \;sin\theta d\theta \;d\phi dA=0.25R_{o}\pi^{2}D^{2}\sin^{2}\theta_{o}$$

where $\theta_{0}$ is the light divergence angle and $R_{o}$ is the source radiance. Radiance is conservative in any optical system given negligible reflection losses or scattering as in our case, where very few optical components are used. In the case of a Lambertian source, the radiance is independent of the viewing angle or the position of the source. In the case of a small divergence angle, $\sin\theta_{o}\sim \theta_{o},$ the product of the area and the divergence angle is called the throughput, denoted as TP, which varies throughout the system depending on the spot diameter D and $\theta_{o}$. For simplification, the constants are omitted and $TP=D^{2}\theta_{o}^{2}$.

To avoid chromatic aberrations (mainly due to a material’s dispersive refraction), off-axis parabolic mirrors are used instead of lenses, as depicted in Figure 2a. The off-axis parabolic mirrors are used to collimate light at the source side and refocus light at the spectrometer side. The off-axis parabolic mirrors have an acceptance angle < 0.5 rad (focal length ≥ half the diameter).

Filament sources have a big throughput compared to that of the input of the MEMS spectrometer. The divergence angle is big (in other words, it is >0.5 rad). This means that the entire area of the mirror (diameter of the mirror is $D_{m}$) will be illuminated. Thus, light throughput from the collimating mirror is $TP_{1}=D_{m}^{2}\theta_{1}^{2}$, where $\theta_{1}$ is the divergence angle of the collimated light, as depicted in Figure 2a.

Light received by the other mirror is given by $D_{m}\theta_{2}$, where $\theta_{2}$ is the divergence angle of the light received by the mirror, and it is equal to $\frac{0.5D_{m}}{L}$, where L is the path length. Thus, the light throughput received by the second mirror is given by $TP_{2}=0.25D^{4}/L^{2}$. It is obvious that on increasing the path length L, the transmission, given by $TP_{2}/TP_{1}$, will degrade. On the other side, the spectrometer has a numerical aperture $\theta_{s}$ and the diameter of the input fiber is $D_{s}$. Such a throughput is much smaller than the source throughput or even $TP_{1}$. There is a range of path lengths from 0 to $L_{max}$ at which the power received by the spectrometer is constant. $L_{max}$ holds when the light throughput $TP_{2}$ of the second mirror is equal to that of the spectrometer. Thus, $L_{max}$ is given by

(2) $$L_{max}=\frac{0.5D^{2}}{\theta_{s}D_{s}}$$

In order to fit the received power within the acceptance of the spectrometer, the optimum focal length of the mirror should satisfy $\theta_{s}=0.5D_{m}/f_{opt}$ or $f_{opt}=\frac{0.5D_{m}}{\theta_{s}}$. For the MEMS spectrometer, this value is much larger than the mirror diameter ($\theta_{s}$ is about 0.05 rad). However, one is restricted to available off-the-shelf components that are comparable to the mirror diameter (i.e., 2D, 3D). Using off-the-shelf mirrors with a focal length $f_{m}$ shorter than the $f_{opt}$ means that the divergence of the light received by the spectrometer is $\theta_{3}>\theta_{s},$ which directly means that the imaged spot at the spectrometer input will be of a diameter $D_{3}<D_{m}$ (because at $L_{max}$, $D_{m}\theta_{2}=D_{s}\theta_{s}=D_{3}\theta_{3}$ and $D_{3}=\frac{f_{m}}{f_{opt}}D_{s}$). Thus, in such a case, the maximum path length should be decreased by a factor $\frac{f_{m}}{f_{opt}}$ to increase the percentage of received power by ${\left(\frac{f_{m}}{f_{opt}}\right)}^{2}$ increasing $D_{3}$ back to $D_{s}$. Thus, the maximum path length of a given mirror of diameter $D_{m}$ and focal length $f_{m}$ should be modified to be

(3) $$L_{max}=\frac{f_{m}D_{m}}{D_{s}}$$

Thus, the maximum path length is increased by the product of both the focal length and the diameter of the parabolic mirror. The insertion loss (*IL*) of the coupling system (two parabolic mirrors of diameter 50.8 mm and focal length 152.4 mm) versus distance was simulated using a ray-tracing software (ZEMAX OpticStudio software). The result is shown in Figure 2c. The *IL* around 26 m was −3.7 dB; it is ascribed to optics aberrations, which degrade the *IL*. For a distance of 38 m, the corresponding losses were −7dB.

### 3.2. District-Scale Experimental Study in Sense City

**Figure 4:**
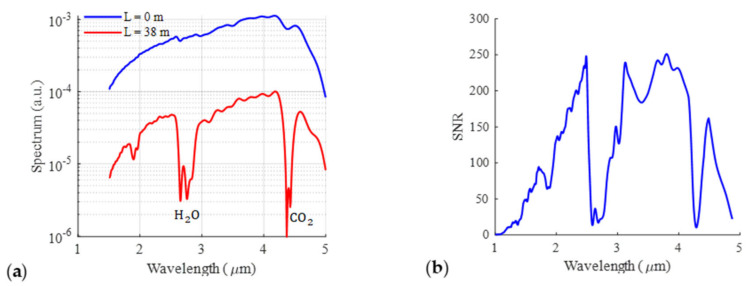
(**a**) Measured spectrum at a distance of *L* = 0 m and 38 m. (**b**) The measured signal-to-noise ratio (SNR) at a distance of *L* = 38 m and a resolution of 80 cm^−1^. The average measurement time is 1 min.

The raw spectrum measured by the FTIR spectral sensor is plotted in Figure 4a and compared to the corresponding spectrum measured at *L* = 0 m. The spectrum’s shape is the same except for the range around the wavelengths of 1.87–2.7 μm and 4.25 μm, corresponding to absorption by water vapor (H_2_O) and by carbon dioxide (CO_2_), respectively. The signal-to-noise ratio (SNR) was measured at a distance of 38 m using a spectral resolution of 80 cm^−1^ and an average measurement time of 1 min. The SNR was calculated using a 100%-line method. The method is based on acquiring a series of successive measurements. The 100% lines are calculated by dividing each two consecutive measurements. Calculating the average across these lines, the SNR is obtained. It is shown in Figure 4b. The detection limits of different gases can be calculated using this SNR curve. The SNR also enables calculation of the noise standard deviation in absorbance from the following equation:

(4) $$\sigma_{A}\left(\lambda \right)=\frac{\log_{10}e}{SNR\left(\lambda \right)\;\sqrt{2}}$$

where λ is the wavelength. Then, one can determine the optimum wavelength $\lambda_{opt}$ for gas detection as the wavelength corresponding to the maximum value of the ratio of absorbance over noise. Then, one can evaluate the minimum detectable concentration from the condition $A\left(\lambda \right)=\sigma_{A}\left(\lambda_{opt}\right)$. Based on that, we were able to determine the detection limit of different gases, and the corresponding results are given in Table 1. The data are compared with those of the reference analyzers. At this stage, it is worthwhile to remember that the Neospectra measures an average value of the gas concentration over the whole distance of 38.1 m, while the gas analyzer measures the local concentration of gas nearby the gas pollutant source at the car gas exhaust, where the concentration is expected to be much higher.

The background effect on the raw measured spectrum is due to the non-idealities in the optical source spectrum, the collimation optics and the spectrometer line shape function. All these effects deform the spectral response and should be removed. Conventionally, this is carried out by measuring a first spectrum in the absence of the sample under test, called the background spectrum. This was not accessible in our case since we were measuring in the open air. This difficulty can be removed using baseline correction methods such as polynomial fit [17], adaptive iteratively weighted penalized least squares [18] or wavelet transform [19]. Polynomial fitting is considered a popular and fast method. This method was applied to all the measured spectra, to also overcome the source stability with time, which leads to a wavelength-dependent shift in the baseline as time evolves.

At the start of the measurement, it is expected that the concentration of gases is almost the same (steady state) in the whole room since no local pollution perturbation has happened for a long time (the chamber is sealed from the outer environment, and the chamber was closed for more than 12 h). Thus, the absorbance value measured by MEMS FTIR at the absorption wavelength of each gas corresponds to the concentration measured by the reference analyzer, also meaning that a rough calibration of the MEMS FTIR can be carried out at this stage by comparison with the data given by the reference analyzer. The absorbance is directly proportional to the gas concentration (absorbance = constant × concentration) according to the Beer–Lambert law. Therefore, the proportional constant can be retrieved from the concentration measured by the analyzer at time 0 and the absorbance value measured by the FTIR. Measurement recordings started at time 0. After 6 h and 10 min, the car, whose position is depicted in Figure 3, was turned on and was turned off at 6 h and 30 min. After 10 min, the car was turned on again for only 5 min.

**Figure 5:**
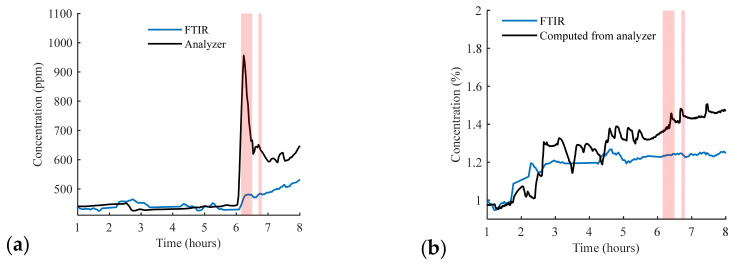

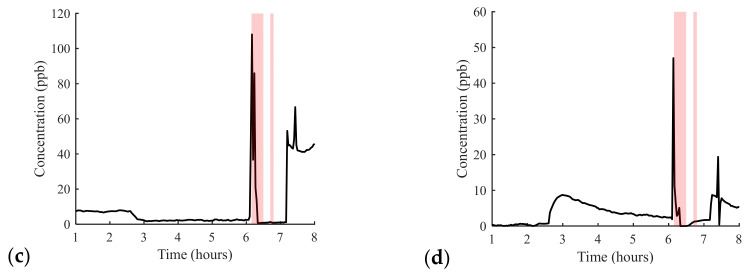
(**a**) Carbon dioxide concentration versus time. (**b**) Water vapor concentration versus time. The red region represents the interval of introducing pollution using car acceleration. (**c**) NO concentration measured by the analyzer. (**d**) NO_2_ concentration measured by the analyzer.

The concentration of the measured carbon dioxide versus time is depicted in Figure 5a. The analyzer measurement is plotted within the same figure for comparison. The translucent red part represents the duration when the car was running (pollution was introduced). A spike in CO_2_ concentration can be observed in the analyzer measurement because the analyzer was placed in close proximity to the car. After the car was turned off, the CO_2_ concentration started to decrease due to its diffusion inside the room. On the other side, the open-path FTIR measured a rapid increase in CO_2_, once the car was turned on, but it was no faster than that of the analyzer and it did not reach the same peak. This is because the FTIR was averaging the concentration over a long distance. After a while, a gradual increase in the carbon dioxide was measured with a slower rate due to the continuous diffusion of carbon dioxide inside the room.

The concentration of water vapor was monitored for a wavelength peak at 1.848 μm in the FTIR measurement. It was compared using the water concentration evaluated from the humidity sensor and the temperature sensor, as depicted in Figure 5b. The saturated water vapor pressure (Pascal) is calculated using the Magnus formula as follows [20]:

(5) $$P_{H_{2}O}{}_{sat.}=0.61exp\left(\frac{17.62T}{243.12+T}\right)$$

where *T* is the temperature in °C and the corresponding water concentration is given by

(6) $$C_{H_{2}O}\left(\%\right)=RH\left(\%\right)*\frac{P_{H_{2}O}{}_{sat.}}{P}$$

where *P* is the total air pressure. The concentrations of NO and NO_2_ measured by the analyzer are plotted in Figure 5c,d, respectively. It is worth mentioning that there was a signal interruption in the analyzer from 6:10 to 7:10, causing a drop in the signal during this period. However, a spike in both gases can be observed once the pollution started.

The detection limits, as summarized in Table 1, show that the MEMS-based setup had a detection limit of water relative humidity (RH) of 0.07%, which is much less than the outdoor typical values (30–50%). For CO_2_, the detection limit was 40 ppb, which is also much less than the typical outdoor values (250–400 ppm). On the other side, the detection limit for NO was much deviated from the typical values. According to that, the presented setup presents a promising solution using open-path measurements surpassing conventional open-path FTIR sensors, which suffer from high cost, bulkiness and heaviness [21,22,23].

### 3.3. Laboratory-Scale Study on Volatile Organic Compounds

To complement the first part of this study, we conducted additional preliminary experiments at the laboratory scale, aiming to assess on the capabilities of our spectral sensor regarding volatile organic compounds (VOCs), which is another important family of indoor gaseous pollutants. An experiment was conducted to determine the lower limit of detection (LLOD) of toluene [24], selected among the BTEX family (benzene, toluene, ethylbenzene, xylenes), known to be among the most harmful of VOCs. Toluene has a strong absorption at 3.27 μm, which can serve to monitor its concentration.

**Figure 6:**
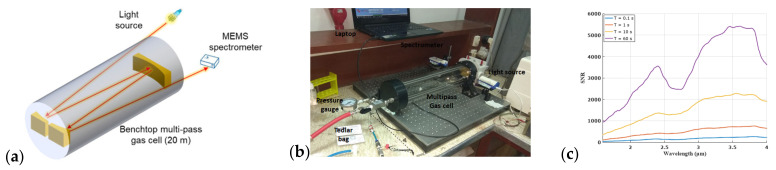
(**a**) Schematic of multi-pass gas cell. (**b**) Experimental setup for measuring toluene (adapted with permission from Ref. [24], A. Fathy et al., 2019, SPIE). Toluene with a given concentration is transferred from the Tedlar gas to the multi-pass gas cell. A light source is used to inject light into the cell and finally detected by the spectrometer. (**c**) Measured SNR for different average times.

The method for assessing gas concentration is displayed in Figure 6a. We inserted light from the source into the multiple-pass gas cell via a collimation lens. The light then made 40 rounds within the gas cell, and a separate lens gathered the output from the cell and directed it back into the MEMS spectrometer. We inserted toluene into the gas cell at various carefully measured concentrations in both the ppm and the ppb ranges, documenting the spectral response at each concentration level.

**Figure 7:**
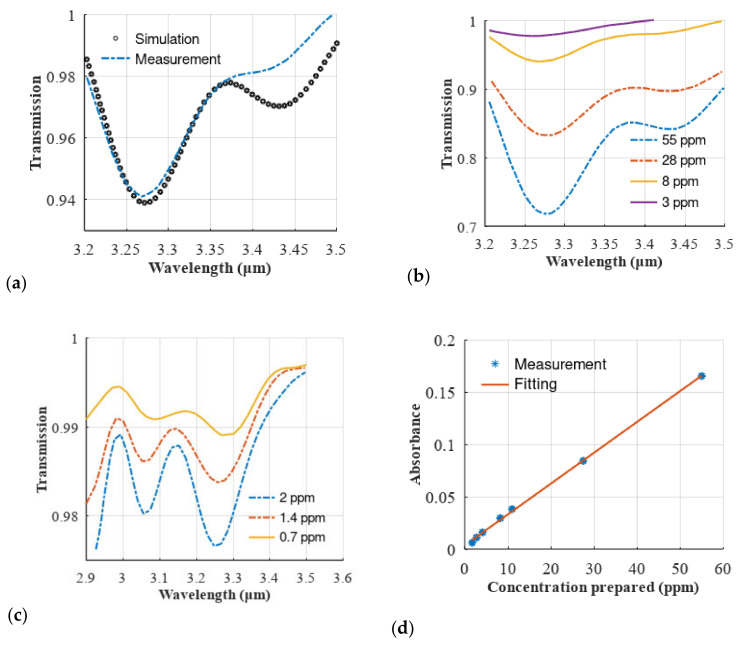
Adapted with permission from Ref. [24], A. Fathy et al., 2019 SPIE. (**a**) Simulated and measured spectrum of toluene at a concentration of 8 ppm, (**b**) measured spectra of toluene at different concentrations in the ppm range (from 8 to 55 ppm), (**c**) measured spectra of toluene at different concentrations in the ppb range (from 700 to 2000 ppb). The spectrum is affected by the side lobes of water absorption, whose relative effect is apparent especially at the lowest concentrations levels of toluene. (**d**) Linearity of measured absorbance versus the corresponding concentrations. The measured points are fitted to a straight line showing good linearity. The regression coefficient is $R^{2}=0.9992$.

We charted a transmission spectrum through 8 ppm of toluene with an 80 cm^−1^ resolution. This measured transmission curve is graphically represented alongside the hypothetical curves in Figure 7a. The recorded data align well with the simulations. Any discrepancies observed after 3400 nm in the curve are likely due to the self-apodization of the MEMS spectrometer, a deviation from the ideal case.

We also charted the transmission spectra for the 3, 8, 28 and 55 ppm concentration levels, as shown in Figure 7b. These curves depict a consistent decrease in the transmission curve with a decreasing concentration. In Figure 7c, we present the transmission curves for 700 and 2000 ppb. At lower concentrations, the residual water in the gas cell produces side lobes that impact the spectrum. These lobes obscure the absorbance of toluene around the 3.27 µm mark.

We investigated the linearity of the system by plotting toluene absorbance against its corresponding concentration *C,* as shown in Figure 7d, using the absorbance formula $A=-\log_{10}T$, where T is the transmittance value at a wavelength of 3.26 μm obtained from the data shown in Figure 7b,c. We then fitted this curve to a straight line, as shown in Figure 7d. This figure demonstrates strong linearity (with a coefficient of determination of R^2^ = 0.9992) across a broad dynamic range.

The system’s signal-to-noise ratio (SNR) was evaluated by filling the gas cell with nitrogen and applying the 100%-line method. We measured the SNR at various average times, as shown in Figure 6c. The SNR, when measured at lambda λ = 3.27 µm, was determined to be 5000:1. This figure corresponds to a standard deviation σ in the absorbance of approximately 10^−4^. Using the curve fitted in Figure 7d, we calculated the minimum detectable concentration as follows:

(7) $$C_{min}=\frac{\sigma }{m}$$

In this equation, m represents the slope of the fitted line. From this, we deduced that the minimum detectable concentration is around 30 ppb.

Coming back to the experiments conducted in Sense City and described in Figure 1 and Figure 2, the very large space allowed the implementation of *open-path absorption spectroscopy* over a large distance of *L* = 38 m. Such a large distance is favorable to reach low detection limits according to the Beer–Lambert Law. On the other side, the latest experiments presented in Figure 6 and Figure 7 also relate to a rather large distance of *L* = 38 m, although these experiments were conducted at the laboratory scale. This was made possible by implementing *multiple-pass gas cell absorption spectroscopy*, which allows operation in a compact space but at the cost of using an expensive accessory: the multiple-pass gas cell. Another major difference between the two methods is that open-path absorption spectroscopy gives the average information about the gas pollutants across the whole propagation distance *L*, while multiple-pass gas cell absorption spectroscopy gives more localized information about the gas pollutants that are inside the cell (which is typically 50× smaller than the propagation distance *L*).

## 4. Conclusions

In conclusion, we presented the potential of a low-cost and compact spectral sensor to monitor multiple gaseous air pollutants simultaneously. The sensor is based on an open-path FTIR gas analyzer that uses an MIR MEMS spectrometer produced using MEMS technologies. The experimental results obtained in a large climatic chamber demonstrated that the proposed sensor is capable of district-scale monitoring of carbon dioxide and water vapor simultaneously, with limits of detection of 40 ppb and 0.07% relative humidity (RH), respectively. This first set of measurement was in good agreement with the reference analyzers. Additionally, laboratory-scale measurements showed promising results for the detection of VOCs as they revealed a limit of detection of 30 ppb for toluene. Overall, the proposed spectral sensor has the potential to be a key enabling technology for ubiquitous pollution monitoring due to its low cost and compactness. While the performance of the sensor is still not yet sufficient for NO_2_, it is already very good for most applications requiring the measurement of CO_2_, RH and VOCs. Furthermore, the proposed sensor has the potential to differentiate multiple VOCs, which is not possible with commercial sensors that only provide information about total VOCs. The proposed sensor is a promising tool to address the growing concern of air pollution and improve air quality. Nevertheless, the sensor has to be evaluated regarding its ability to withstand harsh environments although it was already exposed to relative humidity (RH) levels ranging from 40% to 63% and exposed to car exhaust pollutants that contain not only gaseous pollutants but also solid-state microparticles.

## Figures and Tables

**Table 1:** Detection limit of different gases in case of the open-path FTIR and the reference analyzers in Sense City, compared to outdoor typical values.

Gas	Open-Path FTIR		Reference Analyzer	Outdoor
	Wavelength (nm)	Detection Limit (1σ)	Detection Limit (1σ)	Typical Values
Relative humidity (RH)	2700	0.07%	-	30–50%
CO	4596	800 ppb	25 ppb	50–120 ppb
${\mathrm{CO}}_{2}$	4256	40 ppb	-	250–400 ppm
NO	2677	50 ppm	0.2 ppb	0.1–20 ppb
${\mathrm{NO}}_{2}$	3446	1 ppm	0.2 ppb	40–500 ppb

## Data Availability

Data available upon reasonable request.
